# A Novel Monoclonal Antibody Targets Mucin1 and Attenuates Growth in Pancreatic Cancer Model

**DOI:** 10.3390/ijms19072004

**Published:** 2018-07-09

**Authors:** Guang Wu, Sony Maharjan, Dongbum Kim, Jung Nam Kim, Byoung Kwon Park, Heeju Koh, Kyungduk Moon, Younghee Lee, Hyung-Joo Kwon

**Affiliations:** 1 Center for Medical Science Research, College of Medicine, Hallym University, Chuncheon 24252, Korea; wuguang0412@hallym.ac.kr (G.W.); sonymaharjan@gmail.com (S.M.); hahadb@hallym.ac.kr (D.K.); ihoo@hallym.ac.kr (B.K.P.); 2 Department of Microbiology, College of Medicine, Hallym University, Chuncheon 24252, Korea; ads5467@naver.com; 3 Peptron, Inc., 37-24, Yuseong-daero 1628 beon gil, Daejeon 34054, Korea; hjko@peptron.co.kr (H.K.); losig79@peptron.co.kr (K.M.); 4 Department of Biochemistry, College of Natural Sciences, Chungbuk National University, Cheongju 28644, Korea

**Keywords:** Mucin1, monoclonal antibody, pancreatic cancer, antibody therapeutics, targeting

## Abstract

Mucin1 (MUC1) is a highly glycosylated transmembrane protein that plays a crucial role in the lubrication and protection of normal epithelial cells. However, MUC1 has emerged as a potential target for cancer therapy because it is overexpressed and functions in several types of cancers. Recently, we produced a monoclonal antibody (the anti-hMUC1 antibody) specific to the extracellular region of the MUC1 subunit MUC1-C to evaluate the utility of using anti-MUC1 antibodies in pancreatic cancer models. The anti-hMUC1 antibody recognized the MUC1-C protein in pancreatic cancer cells. Based on immunostaining and confocal image analyses, the anti-hMUC1 antibody initially bound to the cell membrane then was internalized in cancer cells that express MUC1. The anti-hMUC1 antibody suppressed epidermal growth factor (EGF)-mediated extracellular signal–regulated kinase (ERK) phosphorylation and cyclin D1 expression. When the anti-hMUC1 antibody was injected into a xenograft mouse model and traced using an in vivo imaging system, we observed that the anti-hMUC1 antibody was localized to MUC1-expressing pancreatic tumors. Importantly, the anti-hMUC1 monoclonal antibody suppressed pancreatic tumor growth in mice. According to immunohistochemistry analysis using a pancreatic cancer tissue array and the anti-hMUC1 antibody, MUC1 was highly expressed in human pancreatic cancer tissues compared to normal tissues. Therefore, we conclude that the anti-hMUC1 antibody specifically targets MUC1 and suppresses its function in pancreatic cancer in vitro and in vivo and can be further developed as a promising targeted therapy to treat pancreatic cancer.

## 1. Introduction

Pancreatic cancer is one of the predominant causes of cancer-associated death in humans worldwide [1,2]. On an average, below 8% of patient with pancreatic cancer survives for 5 years after diagnosis [2]. Little progress has been made in improving the survival rate of pancreatic cancer, mainly due to the fact that pancreatic cancer is often undetected until late stages [1,2]. For the pancreatic cancer that is able to be diagnosed at an early stage (stage IA and IB), survival rate is improved with surgery and chemotherapy. The average survival rate five years after surgery is still only 27–31%; 67–71% of patients survive one year and 45–50% of patients survive two years [3].

Epithelial surfaces form a mucous layer, which functions to shield and protect organ surfaces. An important component in the protection and lubrication of organs is the high molecular weight glycoprotein mucin, which is expressed specifically by epithelial cells [4]. Mucin1 (MUC1) is a heterodimeric glycoprotein containing an extracellular N-terminal domain (MUC1-N) and a C-terminal domain (MUC1-C). MUC1-C has an extracellular domain, transmembrane domain and cytoplasmic tail and thus spans the cell membrane [5,6]. Mucin protein contains tandem repeat structures known as PTS domains, which contain numerous proline, threonine and serine amino acids [7]. The diversity of mucin genes is derived from variations in the PTS domains. The serine and threonine residues are heavily glycosylated with *O*-linked oligosaccharides [5,7]. Mucins are divided into either membrane-bound or secreted classes based on their physiological and structural characteristics [8]. MUC1, MUC3A, MUC3B, MUC4, MUC11, MUC12, MUC13, MUC15, MUC16, MUC17, MUC20 and MUC21 are members of the membrane-bound transmembrane family of mucins, while MUC2, MUC5AC, MUC5B, MUC6 and MUC19 are secreted mucins [5]. In normal tissue, mucins are heavily glycosylated and are localized to the apical surface of epithelial cells. However, in cancerous tissues, mucins are often hypoglycosylated and dispersed evenly throughout the cell surface [5,7], indicating a potential cancer-promoting role for mucins.

Mucins are known to be present in pancreatic cancer, with elevated expression of multiple MUC family including MUC1 [5]. MUC1 overexpression specifically has been linked to tumor progression, invasion and metastasis in breast and pancreatic cancer cells [9,10,11]. In addition, MUC1 expression is associated with resistance to anti-cancer drugs, presumably leading poor patient prognosis [12]. Studies have shown increased expression of MUC1 in pancreatic cancer in contrast to the low expression levels observed of MUC1 in the luminal surfaces of a normal control pancreases [5,13,14]. We are thus interested in blocking MUC1′s role in pancreatic cancer.

MUC1 may interact with other known growth factor receptors including EGF receptor (EGFR) to promote pancreatic cancer. Previous studies have demonstrated aberrant expression of both MUC1 and EGFR in pancreatic cancer [15,16]. EGFR is a receptor tyrosine kinase known to be involved in multiple cancers. EGFR-mediated signaling leads to activation of multiple pathways, including the mitogen-activated protein kinase (MAPK) and Akt pathways, to enhance cell survival and proliferation. Blocking EGFR signaling by monoclonal antibodies or tyrosine kinase inhibitors induces apoptosis and reduces proliferation in vitro and in cancer xenograft models [17,18]. Targeting of MUC1 inhibits EGFR signaling and reduces EGFR-mediated cancer growth [19].

To our knowledge, minimal progress has been made in the development of potential antibodies to use in the treatment of pancreatic cancer. However, we developed a MUC1-C specific monoclonal antibody (anti-hMUC1 monoclonal antibody) that decreases proliferation of breast cancer cells in vitro and efficiently targets MUC1 in breast tumor in a xenograft mouse model [20]. In this study, we have tested the anti-hMUC1 antibody in pancreatic cancer cells. Here, we observe that the anti-MUC1 monoclonal antibody specifically recognizes MUC1 in both the cell surface and the cytoplasm of pancreatic cells. The anti-hMUC1 monoclonal antibody effectively targeted tumor-specific MUC1 without affecting normal tissue. Importantly, our antibody significantly attenuated the growth of tumors in a pancreatic cancer cell xenograft mouse model. Therefore, this study suggests anti-hMUC1 monoclonal antibody may be a promising novel therapeutic for the treatment of pancreatic cancer.

## 2. Results

### 2.1. Immunoreactivity and Internalization of the Anti-hMUC1 Monoclonal Antibody in Multiple Pancreatic Cancer Cell Lines

**Figure 1:**
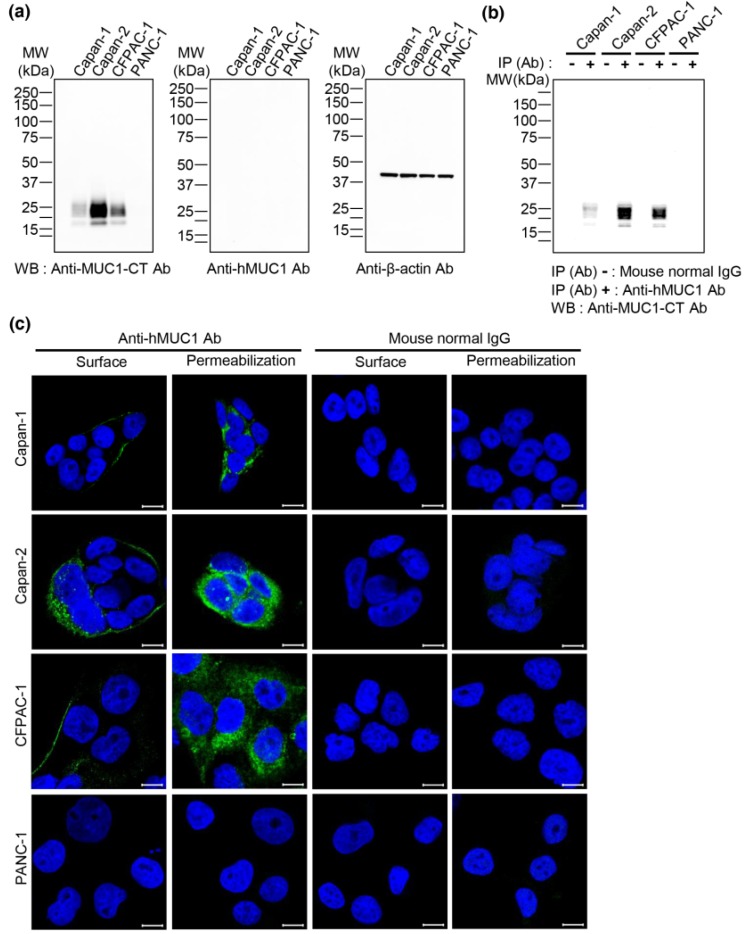
Immunoreactivity of anti-hMUC1 monoclonal antibody in pancreatic cancer cells. (**a**) Lysates from the pancreatic cancer cell lines Capan-1, Capan-2, CFPAC-1 and PANC-1 were analyzed by western blotting using anti-MUC1-CT, anti-hMUC1 and anti-β-actin antibodies. (**b**) Capan-1, Capan-2, CFPAC-1 and PANC-1 cell lysates were immunoprecipitated with mouse normal IgG or anti-hMUC1 monoclonal antibody followed by western blotting using anti-MUC1-CT antibody. (**c**) Capan-1, Capan-2, CFPAC-1 and PANC-1 cells were fixed with 4% paraformaldehyde and incubated with mouse normal IgG or anti-hMUC1 monoclonal antibody on ice to identify MUC1-C on the membrane surface of the cells (surface). On the other hand, to identify MUC1-C in the intracellular region, the cells were fixed, permeabilized and incubated with mouse normal IgG or anti-hMUC1 monoclonal antibody at room temperature (permeabilization). Then, the cells were stained with Alexa 488-conjugated secondary antibody. Hoechst 33258 was used for staining nuclei. Images were captured using confocal microscopy. Scale bars, 10 µm. These results are representatives of three independent experiments.

To examine if the anti-hMUC1 monoclonal antibody recognizes MUC1 protein in pancreatic cancer cells, we performed western blot analysis with lysates from Capan-1, Capan-2, CFPAC-1 and PANC-1 pancreatic cancer cell lines. A commercially available anti-hMUC1-cytoplasmic tail (CT) antibody could detect MUC1 protein in Capan-1, Capan-2 and CFPAC-1 cells but not in PANC-1, while the anti-hMUC1 monoclonal antibody did not detect MUC1 protein (Figure 1a). We therefore used the PANC-1 cell line as a negative control throughout this study. To further evaluate whether the anti-hMUC1 monoclonal antibody recognizes endogenous MUC1-C in the native state, immunoprecipitation of the cell lysates was conducted using anti-hMUC1 monoclonal antibody followed by immunoblot analysis. The anti-hMUC1 monoclonal antibody immunoprecipitated MUC1 protein in the native form in Capan-1, Capan-2 and CFPAC-1 cells (Figure 1b). Furthermore, immunofluorescence staining of MUC1 was performed after incubation with anti-hMUC1 monoclonal antibody. The anti-hMUC1 monoclonal antibody did detect MUC-1 protein at the membrane and in the cytoplasm of Capan-1, Capan-2 and CFPAC-1 cells (Figure 1c).

**Figure 2:**
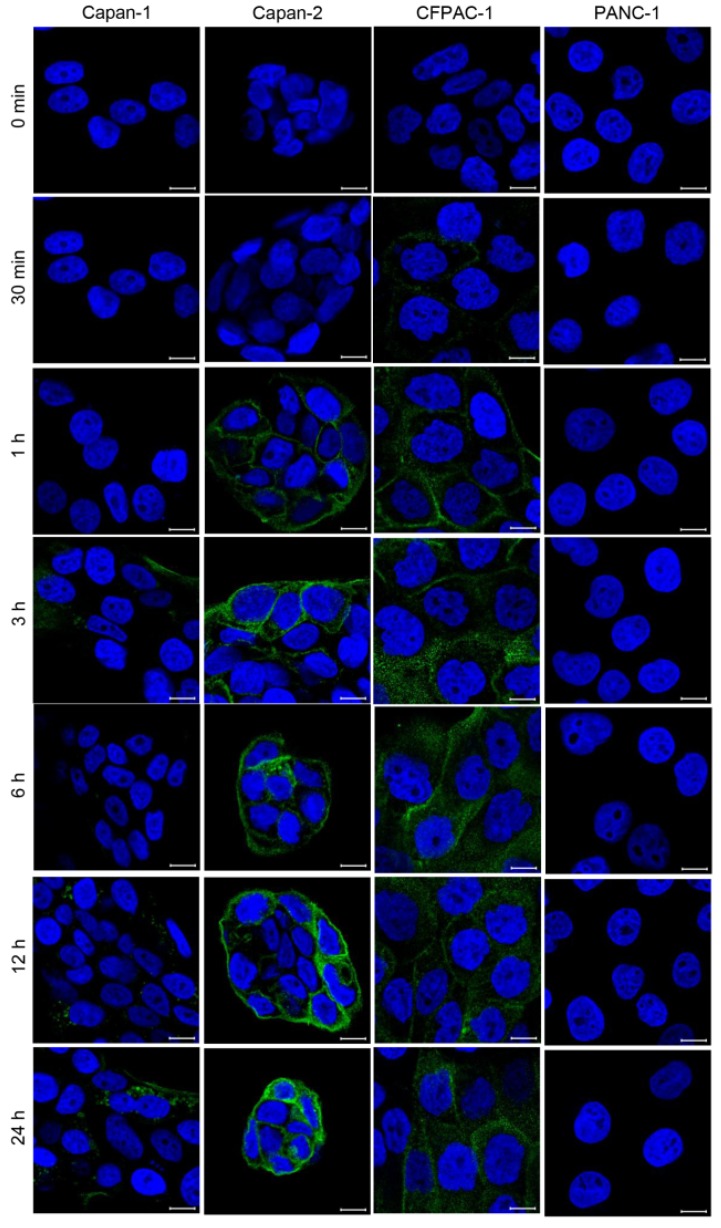
Time-dependent internalization of anti-hMUC1 monoclonal antibody into pancreatic cancer cells. Capan-1, Capan-2, CFPAC-1 and PANC-1 cells were incubated with DyLight 488-conjugated anti-hMUC1 monoclonal antibody for various time intervals and analyzed by confocal microscopy. Hoechst 33258 was used for staining nuclei. Scale bars, 10 µm. These results are representatives of three independent experiments.

In addition, we treated the cells with the anti-hMUC1 monoclonal antibody conjugated with DyLight 488 for different periods and examined the localization of the antibody using confocal microscopy. The anti-hMUC1 monoclonal antibody was effectively internalized in Capan-1, Capan-2 and CFPAC-1 cells in a time-dependent manner (Figure 2). These results suggest that the anti-hMUC1 monoclonal antibody efficiently recognizes endogenous MUC1-C protein in pancreatic cancer cell lines and that the antibody can be internalized after targeting MUC1 protein in pancreatic cells.

### 2.2. Suppression of EGF-Mediated ERK Phosphorylation and Cyclin D1 Expression by Anti-hMUC1 Monoclonal Antibody

**Figure 3:**
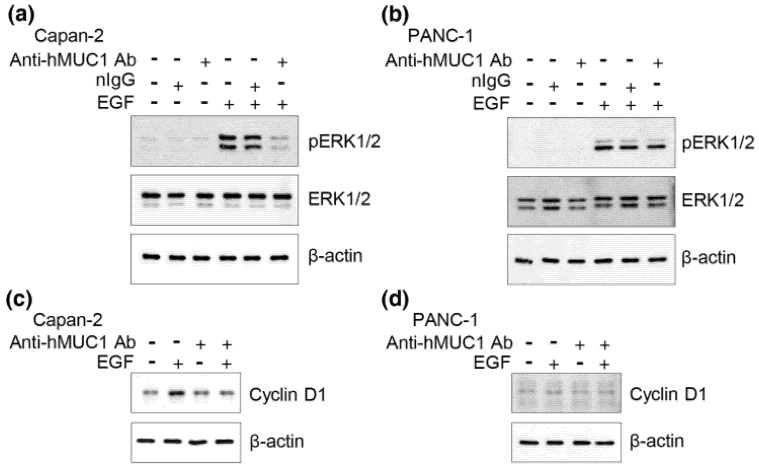
Inhibition of EGF-induced ERK phosphorylation and cyclin D1 expression by anti-hMUC1 monoclonal antibody. (**a**) Capan-2 and (**b**) PANC-1 cells were pretreated with 10 µg/mL anti-hMUC1 monoclonal antibody (anti-hMUC1 Ab) for 6 h followed by stimulation with 10 ng/mL EGF for 5 min. Cell lysates were separated by SDS-PAGE and western blot analysis was performed with anti-phospho-ERK, anti-ERK and anti-β-actin antibodies. (**c**) Capan-2 cells and (**d**) PANC-1 cells were treated with 10 µg/mL anti-hMUC1 monoclonal antibody for 6 h followed by treatment with 10 ng/mL EGF and incubated for 24 h. Cell lysates were separated by SDS-PAGE and western blot analysis was performed with anti-cyclin D1 and anti-β-actin antibodies. These results are representatives of three independent experiments.

MUC1 is known to interact with EGFR and facilitate EGF-mediated cascades [19,21]. ERK phosphorylation is an early event in EGF signaling which further regulates various downstream signals, including expression of cyclin D1. Cyclin D1 promotes cell cycle progression and tumor cell growth [21]. To determine whether the anti-hMUC1 antibody can interrupt EGF-mediated signaling, we pretreated Capan-2 and PANC-1 cells with the anti-hMUC1 monoclonal antibody, followed by EGF treatment. The results from western blot analysis determined that EGF treatment enhances ERK phosphorylation and cyclin D1 expression in Capan-2 cells (Figure 3a,c). However, pre-treatment with the anti-hMUC1 monoclonal antibody significantly reduced ERK-phosphorylation (Figure 3a) and cyclin D1 expression in response to EGF in Capan-2 cells (Figure 3c). In contrast, EGF treatment enhances ERK phosphorylation but there was no induction of cyclin D1 in PANC-1 cells (Figure 3b,d). Furthermore, pre-treatment with the anti-hMUC1 monoclonal antibody had no effect on ERK phosphorylation and cyclin D1 expression in PANC-1 cells. To investigate the effect of the anti-hMUC1 monoclonal antibody on the growth of MUC1 expressing cells, we checked proliferation of PANC-1 cells and Capan-2 in the presence or absence of the antibody. However, there was no suppressive effects in both cells. Furthermore, proliferation of PANC-1 cells and Capan-2 cells was not affected by the anti-hMUC1 monoclonal antibody even after stimulation with EGF. The anti-hMUC1 monoclonal antibody did not induce apoptosis of the cells, either. Taken together, the anti-hMUC1 monoclonal antibody suppressed EGF-mediated signaling in MUC1 expressing pancreatic tumor cells even though we could not detect its growth-suppressive effect in vitro.

### 2.3. Localization of Anti-hMUC1 Monoclonal Antibody in Pancreatic Xenograft Tumors

**Figure 4:**
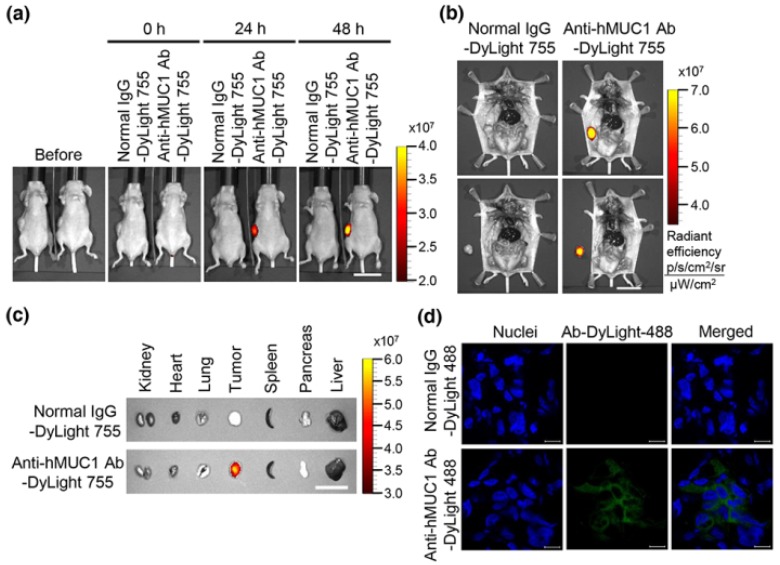
Biodistribution of anti-hMUC1 monoclonal antibody in a xenograft mouse model. BALB/c nu/nu mice were subcutaneously inoculated with Capan-2 cancer cells to allow tumor formation. (**a**) DyLight 755-labeled normal IgG (5 mg/kg) or DyLight 755-labeled anti-hMUC1 monoclonal antibody (5 mg/kg) were intravenously injected into mice, followed by acquisition of whole body fluorescent images at 0, 24 and 48 h using in vivo imaging system. (**b**) Upper panel: The localization of the antibody in the tumor region intact to mice. Lower panel: The localization of the antibody in the tumor region after dissection from the same mice. (**c**) Tumor tissues and organs were isolated and analyzed for the antibody distribution. Scale bars, 2.5 cm (**a**–**c**). (**d**) Confocal images of tumor sections obtained from mice intravenously injected with DyLight 488-labeled normal IgG or anti-hMUC1 monoclonal antibody. The frozen tissues were cut into slices using a cryostat and stained with Hoechst 33258 for nuclei. The mounted samples were examined with confocal microscopy. Scale bars, 10 µm (**d**). The images are representative of data from 3 sets of mice.

To determine the potential utility of the anti-hMUC1 monoclonal antibody to target pancreatic tumors, we established a xenograft mouse model using Capan-2 cells. We injected DyLight 755-labeled normal IgG as a control or the anti-hMUC1 monoclonal antibody into Capan-2 tumor-bearing mice intravenously through the tail vein. The distribution of the labeled antibody was quantified by measuring the total flux (photons/s) of fluorescence at 0, 24 and 48 h using in vivo imaging system. The results revealed localization of the antibody in the tumor only and not in any other organs (Figure 4a,b, Supplementary Figure S1a,b). When the tumor tissue and organs were removed, the anti-hMUC1 monoclonal antibody was localized only in tumor tissue, without affecting other vital organs (Figure 4c, Supplementary Figure S1c). The tumors isolated from mice were sectioned and analyzed by confocal microscopy. Tumor sections obtained from mice injected with the DyLight 488-labeled anti-hMUC1 monoclonal antibody had strong signals, whereas those from control IgG injected mice had no staining, indicating that the binding of the anti-hMUC1 antibody is tumor specific (Figure 4d). These results imply that anti-hMUC1 antibody specifically targets pancreatic cancer in a mouse xenograft model.

### 2.4. Inhibition of Pancreatic Xenograft Tumor Growth by the Anti-hMUC1 Monoclonal Antibody

**Figure 5:**
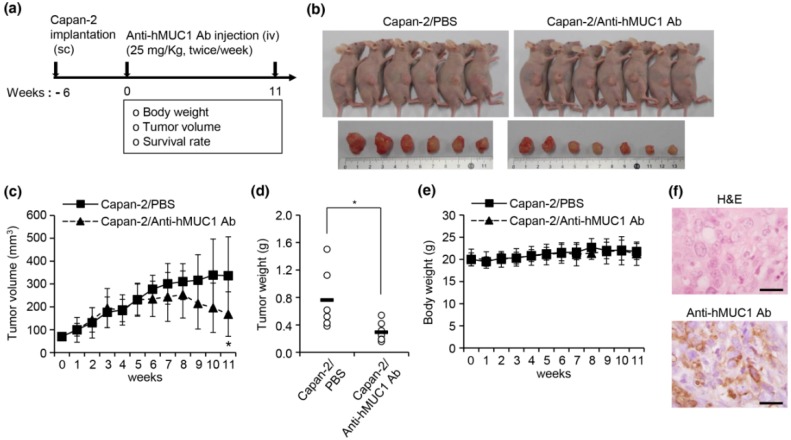
Effect of anti-hMUC1 monoclonal antibody on pancreatic tumor growth in a xenograft mouse model. A mouse xenograft model was established by injection of Capan-2 cells in BALB/c nu/nu mice. Once the tumors reached 80 mm^3^ in volumes, PBS or anti-hMUC1 monoclonal antibody (25 mg/Kg) was intravenously injected into mice. Tumor growth was observed for 11 weeks (*n* = 8). (**a**) Experimental schedule. (**b**) Images of mice bearing tumors and isolated tumors from mice. (**c**) Tumor volumes and (**d**) individual tumor weights for each treatment group. (**e**) Individual body weights for each treatment group. (**f**) Histology of tumor tissue was observed by staining with hematoxylin and eosin (H&E, upper panel). Immunohistochemical analysis of tumor tissue was performed with anti-hMUC1 monoclonal antibody (lower panel). Scale bars, 100 µm. * *p* < 0.05.

To examine the efficacy of the anti-hMUC1 monoclonal antibody in decreasing pancreatic tumor growth in vivo, we utilized our established xenograft mouse model and evaluated the growth of tumors after injection of the antibody into mice. BALB/c nu/nu mice were subcutaneously injected with Capan-2 cells in the dorsal right flank and allowed to grow until the tumor reached 80 mm^3^. The mice were intravenously injected through the tail vein with PBS or anti-hMUC1 monoclonal antibody. The growth of tumor tissues was very slow in mouse xenograft model for pancreatic cancer and the therapeutic effect of the antibody was detected only in the later stage when they already received 16 doses of antibody injection. In the PBS control group, two out of eight mice died, while only one out of eight mice died in the anti-hMUC1 monoclonal antibody group (Figure 5a). When assessing tumor volume and weight, we discovered that mice treated with the anti-hMUC1 monoclonal antibody displayed reduced growth of pancreatic tumors compared to PBS-treated mice (Figure 5b–d). Furthermore, body weight of the mice was not affected by treatment with anti-hMUC1 monoclonal antibody, indicating that the antibody had no notable side effects (Figure 5e). By immunochemical analysis using the anti-hMUC1 monoclonal antibody, expression of MUC1 in pancreas tumor tissue was observed (Figure 5f). Collectively, these experiments suggest that the anti-hMUC1 monoclonal antibody could attenuate pancreatic tumor growth in a mouse xenograft model. As the antibody did not show growth-suppressive effect in vitro, it is hard to explain the suppressive effect in vivo at this moment.

### 2.5. Expression of MUC1 Protein in Human Pancreatic Cancer Tissue

**Figure 6:**
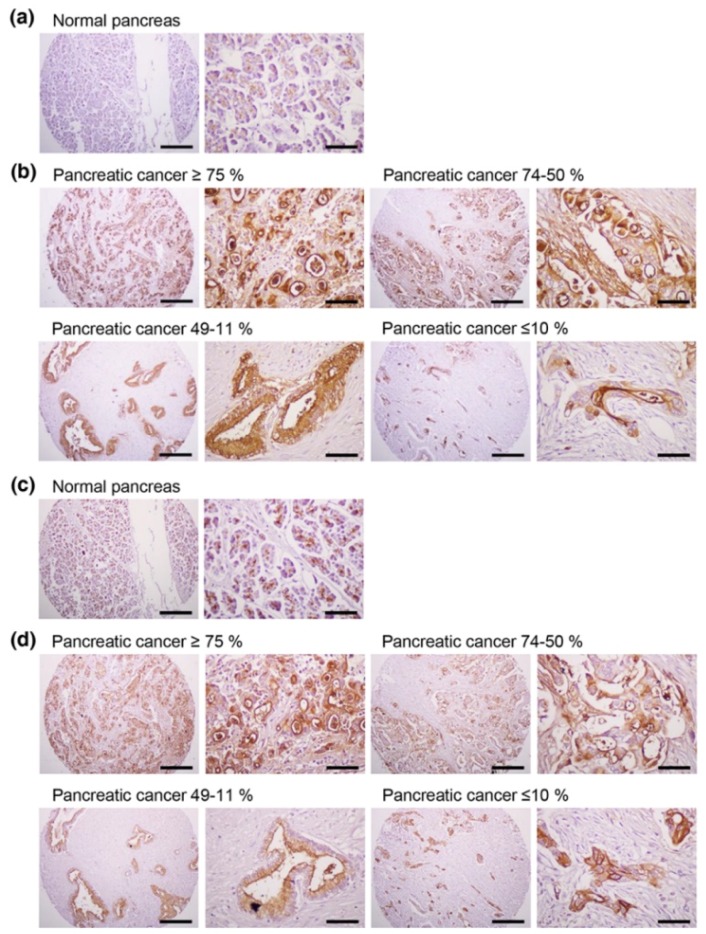
Expression of MUC1 in human pancreatic cancer tissues. Immunohistochemistry of a human pancreatic cancer tissue array was performed with the anti-hMUC1 monoclonal antibody (**a**,**b**) and anti-MUC1-CT2 antibody (**c**,**d**). (**a**,**c**) Normal pancreatic tissue. (**b**,**d**) Pancreatic cancer tissues with ≥75%, 74–50%, 49–11% and ≤10% of tumor cells expressing MUC1. Scale bars; left panel, 100 µm and right panel, 25 µm.

To examine the specificity of the anti-hMUC1 monoclonal antibody in various pancreatic cancer tissues, we stained a human pancreatic cancer tissues array with 33 tumor specimens and matched normal pancreatic tissue for immunohistochemistry (Figure 6a,b). Three percent of the tumor samples had MUC1 expression in ≥75% of tumor cells, 9.1% of samples had 74–50% staining, 48.5% of samples had 49–11% staining and 9.1% of samples had no expression (Table 1). In total, 60.6% of the tumor specimens had MUC1 expression in at least 11% of tumor cells. The levels of MUC1 immunostaining did not correlate with tumor grade or stage. We also stained the same tissue sections of sequential cuts with commercially available anti-hMUC1-cytoplasmic tail (CT) antibody (anti-MUC1-CT2 antibody) (Figure 6c,d). MUC1 expression was similarly detected by anti-hMUC1 monoclonal antibody and anti-MUC1-CT2 antibody. These data indicate that MUC1 is highly expressed in tumors and may have potential as a pancreatic cancer diagnostics marker. This finding also supports the possibility of using the anti-hMUC1 monoclonal antibody in human pancreatic cancer treatment.

## 3. Discussion

Pancreatic cancer is a difficult disease to treat because many patients remain undiagnosed until their cancer is advanced [2,22]. Despite extensive efforts to develop pancreatic cancer therapies, there has been no improvement for decades. The most difficult hurdle to treating pancreatic cancer is the dearth of early symptoms and therefore the late diagnosis of advanced disease [22]. Another reason pancreatic cancer therapies remain often ineffective is that most therapeutics has focused on chemotherapy with cytotoxic drugs, which have limited effect on overall survival [23,24]. Therefore, there is an immediate need for effective targeted therapy to better treat pancreatic cancer.

We previously developed the anti-hMUC1 monoclonal antibody targeting MUC1-C. The anti-hMUC1 monoclonal antibody specifically targets breast cancer cells but not normal cells [20]. To address the clinical need for improved pancreatic cancer therapy, we here evaluated the anti-hMUC1 monoclonal antibody’s potential utility in treating pancreatic cancer. We determined that the anti-hMUC1 monoclonal antibody can specifically target MUC1-C of pancreatic cancer cells in vitro and in vivo and suppresses the growth of pancreatic tumor xenografts.

The anti-hMUC1 monoclonal antibody immunoprecipitated MUC1 in the pancreatic cancer cell lines Capan-1, Capan-2 and CFPAC-1; however, it was unable to detect MUC1-C by western blot analysis. This indicates the anti-hMUC1 monoclonal antibody’s ability to detect endogenous MUC1-C only when the protein maintains its native structure. In addition, confocal microscopy analysis of Capan-1, Capan-2 and CFPAC-1 cells clearly revealed that the anti-hMUC1 monoclonal antibody recognized MUC1-C at the membrane and in the cytoplasm of cells.

The anti-hMUC1 monoclonal antibody targets the MUC1-C subunit, unlike most current MUC1 antibodies that target MUC1-N [4]. MUC1-N domains are shed from the cell surface and are found freely in the extracellular matrix and blood circulation [7,25], which could result in off-target binding of antibodies designed towards MUC1-N. However, MUC1-C remains on the cell surface and is therefore an ideal antibody target. The efficacy of the anti-hMUC1 monoclonal antibody is improved by its ability to pass through the membrane and enter the cytoplasm of the cell. We have further investigated the internalization of the anti-hMUC1 monoclonal antibody by staining the cells with anti-hMUC1 monoclonal antibody conjugated with DyLight 488 fluorescent dye. Confocal analysis showed that the anti-hMUC1 monoclonal antibody induces the movement of MUC1 protein from the membrane into the cytoplasm effectively in both pancreatic cancer cells and xenograft tumors.

For an antibody to be effective as a targeted therapy, it must be specific to tumor and spare normal tissue. Antibodies such as HMFG-1 and C595 are used for the treatment of cancer in preclinical models but they are not shown to have specific affinity to tumor-associated MUC1-C [26,27]. Treatment with the commercially available anti-MUC1 antibody GP1.4 resulted in the inhibition of proliferation and migration of pancreatic cancer cells by activating the internalization of EGFR which leads to sequestration of surface receptors and repression of ERK phosphorylation [19]. However, its tumor specificity or in vivo efficacy has not been examined.

Our results clearly demonstrate that anti-hMUC1 monoclonal antibody conjugated with DyLight 755 exclusively targets MUC1-C in tumor cells but not in normal tissues. This specificity of our anti-hMUC1 monoclonal antibody could be because it recognizes different posttranslational modifications of MUC1 in normal versus tumor tissue. In normal cells, MUC1 is heavily glycosylated and displays polarized expression on the apical surface of the cells. This modification potentially masks the anti-hMUC1 monoclonal antibody recognition site with bulky carbohydrate chains. However, in tumor cells, MUC1 is hypoglycosylated and dispersed throughout the cell membrane, presenting a better recognition site for the anti-hMUC1 monoclonal antibody to recognize and bind to MUC1-C [25].

In addition to effectively recognizing and binding cellular MUC1-C, the anti-hMUC1 monoclonal antibody significantly attenuated tumor signaling. Importantly, the anti-hMUC1 monoclonal antibody significantly reduced the growth of tumors in a Capan-2 xenograft mouse model. Therefore, the need for clinical trials to evaluate the use of the anti-hMUC1 monoclonal antibody in patients with pancreatic cancer is clear. Our data showing expression of MUC1-C in 60.6% of human pancreatic tumors also highlight the plausibility of using the anti-hMUC1 monoclonal antibody to target pancreatic cancer. Because of the exclusive specificity to tumor cells, our anti-hMUC1 antibody could be developed into a potent pancreatic cancer therapy.

The potency of our antibody could be further enhanced by conjugation with toxic components. Previous studies have demonstrated enhanced toxicity of antibodies to tumor cells when the murine antibody HMFG1 is conjugated with an Yttrium moiety and when the C595 antibody is conjugated with docetaxel [26,27]. Another study shows the effective toxicity of the PankoMab antibody conjugated with the β-amanitin toxin on T47D tumor cells [28].

We have demonstrated that the anti-hMUC1 monoclonal antibody specifically binds tumor MUC1-C in vitro, in xenograft models and in human pancreatic cancer tissue. The anti-hMUC1 monoclonal antibody passes through the membrane and inactivates MUC1 oncogenic signaling. When used in pancreatic cancer xenografts, the anti-hMUC1 monoclonal antibody attenuated tumor growth. Although application of the anti-hMUC1 monoclonal antibody in other cancer therapies has yet to be explored, we propose production of the humanized anti-hMUC1 monoclonal antibody and conjugating the anti-hMUC1 monoclonal antibody with a toxic group to effectively treat the pancreatic cancer patients who present with currently untreatable advanced disease. Surely, we will further confirm the specificity of the humanized anti-hMUC1 monoclonal antibody to MUC1-C on the membrane of pancreatic cancer cells and not to other normal tissues in the future work.

## 4. Materials and Methods

### 4.1. Antibodies

We harvested anti-hMUC1 monoclonal antibodies against the extracellular region of MUC1-C from hybridoma cells, established after immunization of BALB/c mice with rhMUC1-EC192 protein and CpG-DNA co-encapsulated in a phosphatidyl-β-oleoyl-γ-palmitoyl ethanolamine: cholesterol hemisuccinate (DOPE:CHEMS) complex as previously described [20]. The amino acid sequence homology analysis showed that rhMUC1-EC192 protein has 26–39% identity with the corresponding sequences of other mucin family members. The anti-hMUC1 monoclonal antibody was not reactive to mouse MUC1 C-terminal protein which has 58% identity [20]. To detect the cytoplasmic tail region of MUC1 protein in cells by western blotting and immunoprecipitation analyses, commercially available anti-MUC1-CT antibody (anti-MUC1-CT Ab, Catalog No. ab109185, rabbit monoclonal antibody to MUC1) was purchased from Abcam (Cambridge, UK). Anti-β-actin antibody was obtained from Sigma-Aldrich (Saint Louis, MO, USA). Anti-phospho-ERK, anti-ERK and anti-cyclin D1 antibodies were obtained from Cell Signaling Technology (Danvers, MA, USA).

### 4.2. Cell Culture

Human pancreatic cancer cell lines Capan-1 (adenocarcinoma, liver metastasis) and PANC-1 (epithelioid carcinoma) were purchased from Korean Cell Line Bank (KCLB, Seoul, Korea). KCLB characterized the cell lines using DNA fingerprinting analysis, species verification test, mycoplasma contamination test and viral contamination test. Human pancreatic cancer cell lines, Capan-2 (adenocarcinoma) and CFPAC-1 (adenocarcinoma, liver metastasis), were purchased from the American Type Culture Collection (ATCC, Manassas, VA, USA). The cell lines were characterized by the tests for morphology, post-freeze viability, interspecies determination (isoenzyme analysis), cytogenetic analysis, mycoplasma contamination and bacterial and fungal contamination. Capan-1 cells were cultured with RPMI-1640 medium (Thermo Fisher Scientific, Waltham, MA, USA), Capan-2 cells in McCoy’s 5A medium (Thermo Fisher Scientific), PANC-1 cells in Dulbecco’s modified Eagle’s medium (DMEM, Thermo Fisher Scientific) and CFPAC-1 cells in Iscove’s modified Dulbecco’s medium (IMDM, Thermo Fisher Scientific). All cell lines were cultured with 10% fetal bovine serum (FBS, Thermo Fisher Scientific), 100 U/mL penicillin and 100 µg/mL streptomycin and were incubated at 37 °C in 5% CO_2_.

### 4.3. Western Blot and Immunoprecipitation Analysis

Cell lysates from pancreatic cancer cell lines were prepared with cell lysis buffer (20 mM Tris·HCl pH = 8.0, 5 mM EDTA, 150 mM NaCl, 100 mM NaF, 2 mM Na_3_VO_4_, 1% NP-40) and centrifuged at 16,000× *g* at 4 °C for 20 min. Proteins from cell lysates were separated in 4–12% Bis-Tris gradient gel (Thermo Fisher Scientific). The separated proteins were transferred onto nitrocellulose membranes and blocked with 3% BSA in PBST for 1 h at room temperature. The nitrocellulose membranes were incubated with anti-hMUC1-CT antibody, anti-hMUC1 monoclonal antibody, or anti-β-actin antibody overnight at 4 °C. Anti-phospho-ERK, anti-ERK and anti-cyclin D1 antibodies were used for analysis of EGF-mediated signaling. The membranes were treated with horseradish peroxidase-conjugated secondary antibody (Jackson ImmunoResearch, West Grove, PA, USA) and the immune-reactive bands were detected by an enhanced chemiluminescence reagent (Thermo Fisher Scientific) as previously described [20,29]. To investigate whether the anti-hMUC1 monoclonal antibody recognizes MUC1-C in pancreatic cancer cells, immunoprecipitation analysis was performed. Briefly, cell lysates were treated with mouse anti-hMUC1 monoclonal antibody or mouse normal IgG overnight at 4 °C and then incubated with Protein A beads at 4 °C for 1 h. The immunocomplexes were identified by western blotting using the anti-hMUC1-CT antibody.

### 4.4. Confocal Microscopy

To obtain confocal images, pancreatic cancer cell lines were cultured on poly-l-lysine-coated glass cover slips in 12-well culture plates as previously described [30,31]. After cells were cultured for 48 h, cells were fixed with 4% paraformaldehyde for 10 min and mouse anti-hMUC1 monoclonal antibody or mouse normal IgG were treated for 4 h on ice for detection of cell surface MUC1-C. For intracellular staining, cells were fixed with 4% paraformaldehyde, permeabilized with 0.1% Triton X-100, blocked with 3% BSA and stained with anti-hMUC1 monoclonal antibody for 2 h at room temperature. After washing cells with PBST (0.1% Triton X-100 in PBS) containing 1% BSA, cells were stained with Alexa Flour 488-conjugated secondary antibody (Thermo Fisher Scientific) for 1 h. Nuclei were stained with Hoechst 33258 (Thermo Fisher Scientific). The samples were observed by confocal laser scanning microscope system (CLSM, LSM 710, Carl Zeiss, Jena, Germany) [30,31]. To visualize the internalization of the MUC1-anti-hMUC1 monoclonal antibody complex in pancreatic cancer cell lines, the anti-hMUC1 monoclonal antibody was labeled with DyLight 488 according to the manufacturer’s instructions (Thermo Fisher Scientific). The human pancreatic cancer cells were incubated with DyLight 488-labeled anti-hMUC1 monoclonal antibody at 37 °C in 5% CO_2_ for the indicated time periods. Fluorescence signals were observed with CLSM (LSM 710, Carl Zeiss).

### 4.5. Animals

Six-week-old female BALB/c nu/nu mice were obtained from Nara Biotech, Inc. (Seoul, Korea). The mice were maintained under specific-pathogen-free conditions in a controlled environment (20–25 °C, 40–45% humidity, 12-h light/dark cycle; ad libitum access to food and water). All animal experimental procedures are in accordance with the recommendations in the Guide for the Care and Use of Laboratory Animals of the National Veterinary Research & Quarantine Service of Korea. The experimental procedures were approved by the Institutional Animal Care and Use Committee of Hallym University (Permit Number: Hallym2015-81, 17, 02, 2016). The mice were anesthetized under isoflurane (2–3%) inhalation with RC2-Rodent Circuit Controller (Lab Etc Inc. Store, Clayton, MO, USA) and all efforts were made to minimize suffering.

### 4.6. In Vivo Imaging

To analyze the distribution profiles of anti-hMUC1 monoclonal antibody, 5 × 10^6^ Capan-2 cells in 50% Matrigel (PBS/Matrigel, 1:1 *v*/*v*, BD Biosciences, Bedford, MA, USA) were subcutaneously injected into the right flank of six-week-old female BALB/c nu/nu mice. The anti-hMUC1 monoclonal antibody and normal mouse IgG were conjugated with DyLight 755 in accordance with manufacturer’s recommendation (Thermo Fisher Scientific). The mouse normal IgG-DyLight 755 (5 mg/kg) or anti-hMUC1 monoclonal antibody-DyLight 755 (5 mg/kg) was intravenously injected into the mice after tumor volumes averaged 300 mm^3^. The antibody distribution profiles were examined with an in vivo imaging system (IVIS 200; Xenogen Corporation, Hopkinton, MA, USA) at 0, 24 and 48 h. Intracellular localization of the anti-hMUC1 monoclonal antibody in tumor tissues in vivo 2 days after injection with DyLight 488-labeled antibody was monitored with CLSM (LSM 710, Carl Zeiss) as described previously [30,31]. The sensitivity of the assay was adjusted to the extent where autoflorescence may not come up.

### 4.7. Xenograft Mouse Studies

Six-week-old female BALB/c nu/nu mice (*n* = 8) were injected subcutaneously in the dorsal right flank with 5 × 10^6^ of Capan-2 cells in a 50% Matrigel solution (PBS/Matrigel, 1:1 *v*/*v*). After tumor volumes averaged 80 mm^3^, mice were injected intravenously with PBS or anti-hMUC1 monoclonal antibody twice weekly. Tumor volumes were measured for 11 weeks at 7 day intervals. The tumor volumes were calculated as width^2^ × length/2 with calipers in three dimensions. The mice were sacrificed 11 weeks after antibody injection and the tumors were surgically excised and weighed. To observe the histopathology, tumors were isolated, fixed in 4% buffered formalin solution and embedded in paraffin by conventional methods. The tissues were cut into 4 µm thick sections. The specimens were stained with hematoxylin and eosin. To identify the expression of hMUC1, the specimens were stained with anti-hMUC1 monoclonal antibody using standard procedures. Mice were euthanized by CO_2_ inhalation (CO_2_ inhalation was performed with 100% CO_2_ at a fill rate of 10–30% of the chamber volume per min) when the tumor size reached 600 mm^3^, the mice lost >20% of initial body weight exhibited evidence of debilitation, pain or distress, such as a hunched posture, rough hair coat, decreased food consumption, emaciation, inactivity, difficulty ambulating and respiratory problems in accordance with experimental procedures approved by the Institutional Animal Care and Use Committee of Hallym University.

### 4.8. Tissue Array and Immunohistochemistry

To investigate the expression of MUC1-C, we obtained human pancreatic cancer tissue sections (AccuMax Array A207(IV)) from ISU ABXIS (Seoul, Korea) with the approval of the Institutional Review Board in Hallym University (approval number: HIRB-2014-114; approval data: 32 December 2014). This study was performed in accordance with the ethical standards of the Declaration of Helsinki. The tissue sections were stained with the anti-hMUC1 monoclonal antibody (1 μg/slide) or anti-MUC1-CT2 antibody (1 μg/slide) in accordance with standard procedures using the Histostain Plus kit (Invitrogen, Carlsbad, CA, USA). Sections were developed with 3,3-diaminobenzidine (DAB, Thermo Fisher Scientific) and stained with hematoxylin to counterstain (Muto Pure Chemicals, Tokyo, Japan). All images were scanned with a light microscope (Eclipse E-200, Nikon, Tokyo, Japan).

### 4.9. Statistical Analysis

Results are shown as a mean ± standard deviation. Statistical significance of differences between two samples was evaluated using Student’s *t*-test; *p* < 0.05 was considered statistically significant.

## Figures and Tables

**Table 1:** Immunohistochemical analysis of MUC1 expression in pancreatic cancer tissues.

Pancreatic Cancer Tissue Sections (AccuMax Array)	*n*	MUC1 Positive (%)	Number (%) of Cases Expressing MUC1				
			≥75%	74–50%	49–11%	≤10%	Negative (%)
A207(IV)	33	60.6	1 (3.0)	3 (9.1)	16 (48.5)	10 (30.3)	3 (9.1)

Percentages in parentheses were calculated as the number of MUC1-psoitive samples for each quartile, divided by the total number of samples in each tumor type.

## Supplementary Materials

Supplementary materials can be found at http://www.mdpi.com/1422-0067/19/7/2004/s1.

## Abbreviations

MUC1-N	MUC1 N-terminal subunit
MUC1-C	MUC1 C-terminal subunit
EGFR	Epidermal growth factor receptor
MARK	Mitogen-activated protein kinase
ERK	Extracellular signal-regulated kinases
DOPE	Phosphatidyl-β-oleoyl-γ-palmitoyl ethanolamine
CHEMES	Cholesterol hemisuccinate
