# Supplementary material for: A Novel Monoclonal Antibody Targets Mucin1 and Attenuates Growth in Pancreatic Cancer Model
Source: Int J Mol Sci. 2018 Jul 9;19(7):2004. doi: 10.3390/ijms19072004 (PMC6073888; doi:10.3390/ijms19072004)
Supplement: Supplementary file 1 [file ijms-19-02004-s001.pdf]

A novel monoclonal antibody targets mucin1 and attenuates growth in pancreatic cancer model

Guang Wu, Sony Maharjan, Dongbum Kim, Jung Nam Kim, Byoung Kwon Park, Heeju Koh, Kyungduk Moon, Younghee Lee, and Hyung-Joo Kwon

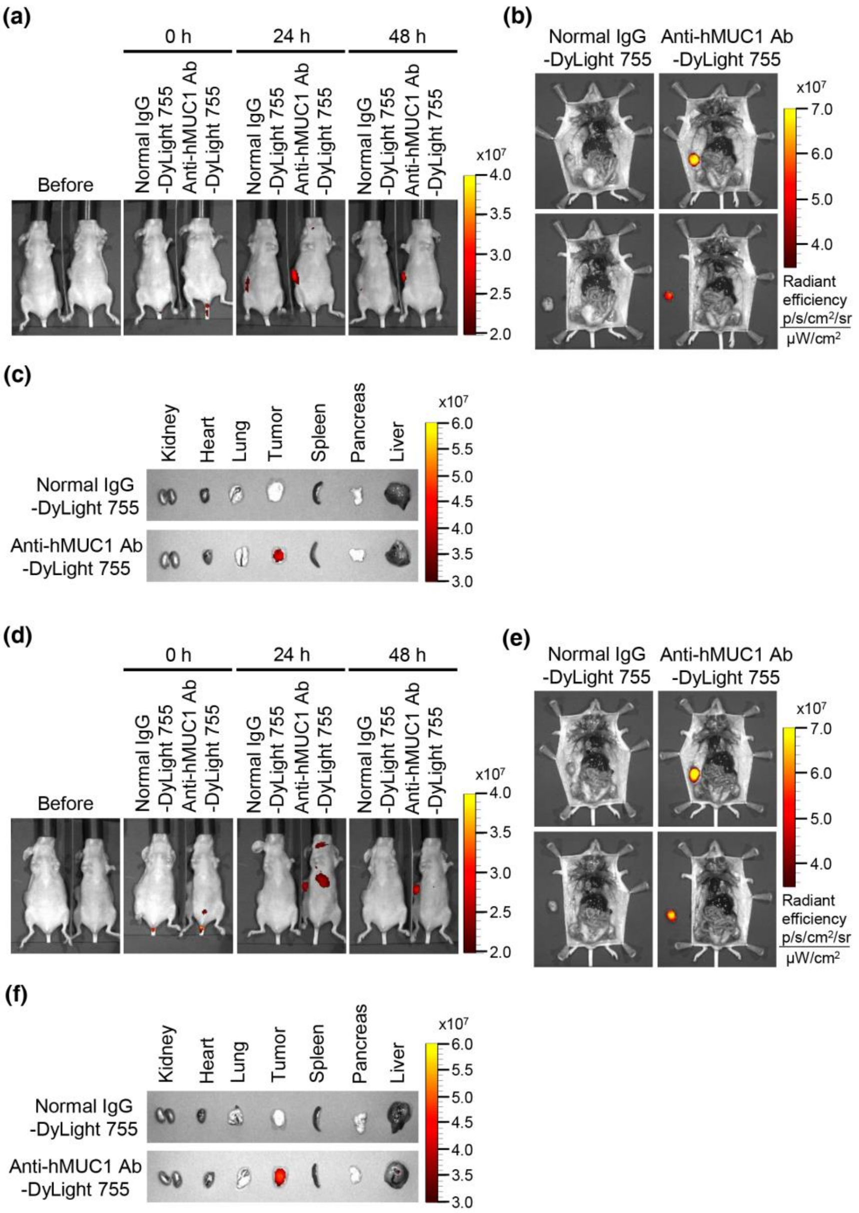

Supplementary Figure S1. Biodistribution of the anti-hMUC1 monoclonal antibody in Capan-2 cell-derived pancreas tumor tissues. BALB/c nu/nu mice were subcutaneously inoculated with Capan-2 cancer cells to allow tumor formation. (a, d) DyLight 755-labeled normal IgG (5 mg/kg) or DyLight 755-labeled anti-hMUC1 monoclonal antibody (5 mg/kg) were intravenously injected into mice, followed by acquisition of whole body fluorescent images at 0, 24, and 48 h using *in vivo* imaging system. (b, e) Upper panel: The localization of the antibody in the tumor region intact to mice. Lower panel: The localization of the antibody in the tumor region after dissection from the same mice. (d, f) Tumor tissues and organs were isolated and analyzed for the antibody distribution. The images are data from 2 sets of mice.
